# Inter-Individual Differences in RNA Levels in Human Peripheral Blood

**DOI:** 10.1371/journal.pone.0148260

**Published:** 2016-02-10

**Authors:** Piotr Chomczynski, William W. Wilfinger, Hamid R. Eghbalnia, Amy Kennedy, Michal Rymaszewski, Karol Mackey

**Affiliations:** 1 Molecular Research Center, Inc. Cincinnati, OH, United States of America; 2 University of Cincinnati, College of Medicine, Cincinnati, OH, United States of America; University of Jaén, SPAIN

## Abstract

Relatively little is known about the range of RNA levels in human blood. This report provides assessment of peripheral blood RNA level and its inter-individual differences in a group of 35 healthy humans consisting of 25 females and 10 males ranging in age from 50 to 89 years. In this group, the average total RNA level was 14.59 μg/ml of blood, with no statistically significant difference between females and males. The individual RNA level ranged from 6.7 to 22.7 μg/ml of blood. In healthy subjects, the repeated sampling of an individual’s blood showed that RNA level, whether high or low, was stable. The inter-individual differences in RNA level in blood can be attributed to both, differences in cell number and the amount of RNA per cell. The 3.4-fold range of inter-individual differences in total RNA levels, documented herein, should be taken into account when evaluating the results of quantitative RT-PCR and/or RNA sequencing studies of human blood. Based on the presented results, a comprehensive assessment of gene expression in blood should involve determination of both the amount of mRNA per unit of total RNA (U / ng RNA) and the amount of mRNA per unit of blood (U / ml blood) to assure a thorough interpretation of physiological or pathological relevance of study results.

## Introduction

The molecular composition of circulating blood reflects the physiological and pathological events in tissues and organs of the human body. The use of peripheral blood in diagnostic applications is therefore desirable for development of biomarkers due to its accessibility and the lower risk associated with its collection, as compared to organ biopsies [1, 2]. Advancements in RNA sequencing have further increased interest in gene expression studies utilizing blood for research and diagnostic purposes [3–5].

Despite the frequent use of blood in gene expression studies, RNA content in human blood has not been thoroughly evaluated. In the literature there is only a limited assessment of blood RNA levels and of RNA recovery from human blood. In previous studies, RNA extracted from human peripheral blood has been reported to be in the range of 1–6 μg of total RNA per ml of blood [5–9]. This amount is markedly lower than the amount of RNA extracted from human blood reported in our preliminary study [10]. That report showed that the average amount of total RNA extracted from the blood of individuals of various age and health conditions was 13.9 μg RNA per ml, with up to 2.7 fold inter-individual differences in total RNA level. Differences in human RNA blood level have been noted before [6, 8]. In the current report, we provide an assessment of RNA levels in the peripheral blood collected from 35 healthy individuals ranging in age from 50 to 89 years. Blood from older adults is frequently analyzed for changes in gene expression when searching for RNA markers that reflect human health status. In this study, we investigated blood RNA level and its inter-individual differences in relation to: white blood cell numbers, DNA level, and the gene expression levels of housekeeping genes in human blood.

## Materials and Methods

### Ethics Statement

Chesapeake Research Review, LLC. CIRBI Protocol # Pro00009509. The IRB specifically approved this study. Participants were provided written informed consent that was signed by the subject and a witness. The informed consent documents were retained. This consent procedure was approved by Chesapeake IRB.

### Blood donors

The study comprised 35 healthy individuals with no clinical history of autoimmune disease. The group included 25 females with an average age of 61.68 years (range from 50 to 82 years) and 10 males with an average age of 65 years (range from 52 to 89 years). The health status of blood donors was evaluated based on a questionnaire containing 53 health-related questions and a complete blood cell (CBC) analysis.

### Blood collection, blood cell count, and sample storage

Blood collection was done by New Horizons Clinical Research, Cincinnati, OH. After an overnight fast, venous blood was drawn into two 10 ml BD Vacutainer tubes with EDTA (K2) as anticoagulant (Becton Dickinson and Company). One tube was used for CBC analysis and the second one for RNA and DNA isolation. The CBC analysis included counts of white blood cell (WBC) types, red blood cells (RBC) and platelets by automated analysis (S1 Table) performed by LabCorp Dublin, OH. Blood samples containing 8–10 ml of blood were transferred from the Vacutainer tubes into pre-weighed bottles containing 16 ml of RNAzol^®^ BD. The resulting blood/reagent mixture was thoroughly shaken to form a homogeneous lysate and stored at -20°C. The blood-RNAzol^®^ BD lysates can be stored at -20°C for at least 1 year with no discernable degradation of RNA.

### RNA and DNA isolation

The isolation of large RNA and small RNA fractions from frozen blood lysates was performed using RNAzol^®^ BD reagent as described in the manufacturer’s brochure (Molecular Research Center, Cincinnati, OH). The large RNA fraction contains high molecular weight RNA (>200 nucleotides), including ribosomal RNA (rRNA), messenger RNA (mRNA) and long noncoding RNA (lncRNA). The small RNA fraction (<200 nucleotides) contains 5S rRNA, tRNA, snoRNA, scaRNA, piRNA and other micro RNAs.

The blood-RNAzol^®^BD lysates were weighed to determine the exact amount of blood in each sample assuming blood specific gravity of 1.06 g/ml [11]. After adjusting RNAzol^®^ BD volume and adding acetic acid, the blood lysates were incubated at room temperature for 10 minutes and mixed with 5% (v/v) of bromochloropropane. The mixture was centrifuged and the supernatant was transferred to a new tube. The bottom phenolic phase was immediately stored at -20°C for future DNA isolation. The RNA-containing supernatant was mixed with 0.35 volume of isopropanol to precipitate the large RNA fraction. The precipitate was isolated by centrifugation. The resulting supernatant was used to isolate the small RNA fraction by precipitation with an additional 0.65 volume of isopropanol. The precipitated large and small RNA fractions were washed in 75% ethanol and dissolved in water. For long-term storage, RNA samples were dissolved in FORMAzol (Molecular Research Center, Inc.) and stored at -20°C. RNA concentration was determined based on optical density (OD) at A_260_ nm, assuming that 1 OD of RNA dissolved in water corresponds to 40 μg RNA/ml, and 1 OD of RNA dissolved in FORMAzol corresponds to 44.9 μg RNA/ml. The average A_260/280_ and A_260/230_ ratios of the large RNA fraction from the 35 samples were 2.07 ± 0.01 and 1.69 ±0.26, respectively. Total RNA content in the peripheral blood was calculated as the sum of the large RNA and small RNA fractions. The isolated RNA samples were analyzed with the Agilent Bioanalyzer RNA Nano 6000 Kit (Agilent Technologies, Inc., Santa Clara, CA).

Genomic DNA was isolated from the frozen phenolic fraction by extraction with guanidine thiocyanate at alkaline pH as described in the RNAzol^®^ BD manufacturer’s brochure.

### Reproducibility of Blood RNA Extractions

For validation of the RNA isolation protocol, we determined the variability of RNA recovery in multiple RNA extractions (n = 12) from a single blood draw. For this study, about 50 ml of peripheral blood was collected from two individuals with low and high blood RNA levels, respectively. Aliquots of 2.5 ml of whole blood were added to 5 ml of RNAzol-BD and the resulting lysates were frozen and used for RNA extraction over a period of several months. RNA yield for these two individuals was 9.37 ± 0.33 and 23.17 ± 0.45 μg of total RNA / ml blood with a respective coefficient of variation (CV) of 3.54% and 1.94%. Thus, for these blood samples, the mean methodological CV associated with extractions of RNA from aliquots of the same blood draw was 2.74%.

### Stability of individual blood RNA level over a 9 month period

**Fig 1:**
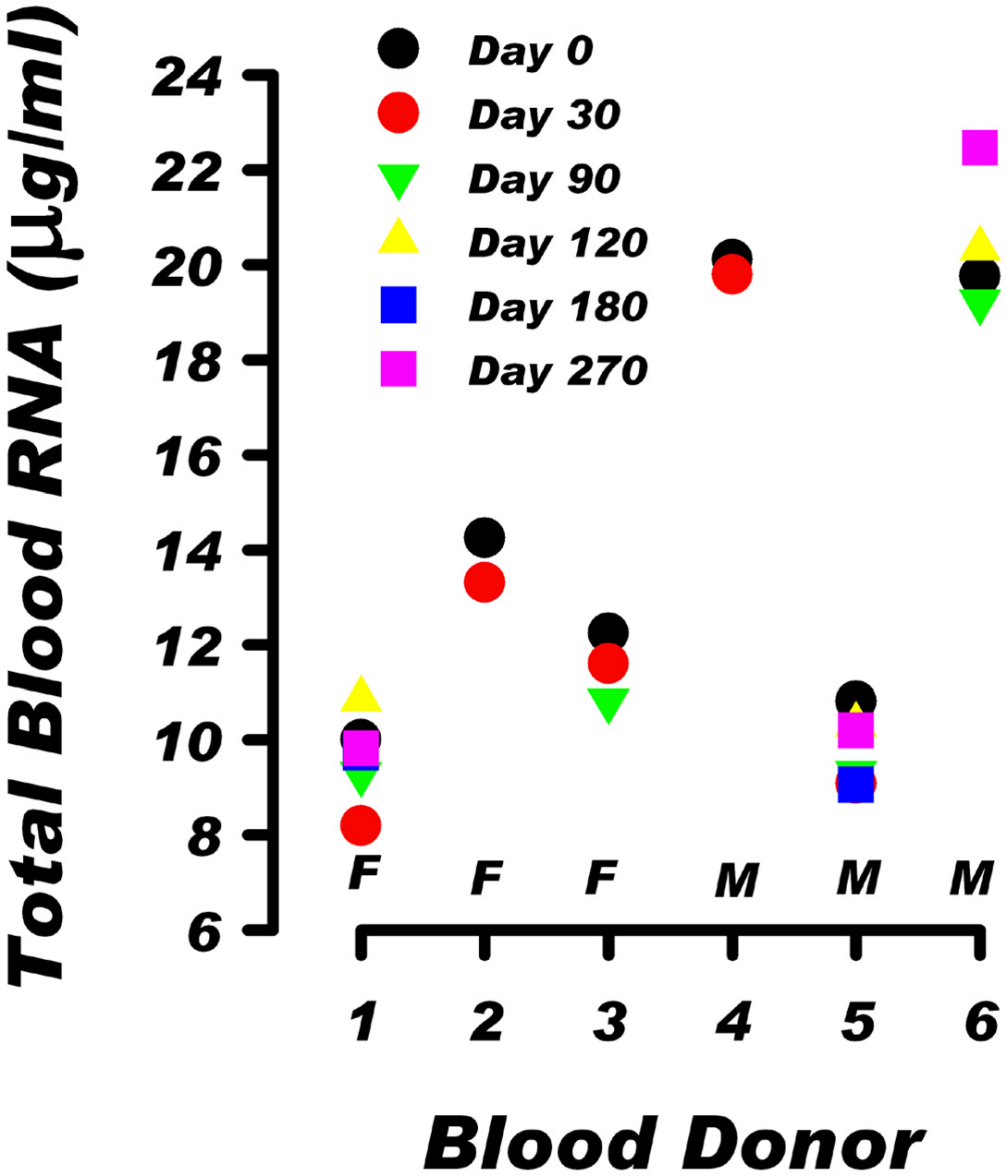
The total RNA level in blood collected over a period of 30 to 270 days from female (F) and male (M) donors. Total RNA was extracted from six blood donors using RNAzol BD as described in the Methods section. The calculated within-individual coefficient of variation of blood RNA level for the six donors was 9.2, 4.8, 6.2, 1.1, 7.6 and 6.9, respectively.

In the preliminary experiment, we examined blood RNA levels, over a 1 to 9 month period, in a group of healthy donors consisting of three women and three men. During this period of time there was no reported change in health status of the donors. Results in Fig 1 demonstrate that in blood from six donors the total RNA level ranged from 9.65 to 20.45 μg /ml. The individual RNA level in blood, whether low or high, was rather stable over time. The mean CV for the group was 5.87%, and the individual CV ranged from 1.13% to 9.15%. Thus, the mean CV of 5.87% for these six samples and the CV of 2.74% for RNA extraction indicate that methodological and/or intra-individual variations in blood RNA level cannot account for the over 2-fold inter-individual differences in the RNA blood level among healthy individuals.

### Reverse Transcription and Quantitative PCR (RT-qPCR)

Reverse transcription was performed with 1 μg of the large RNA fraction using the High-Capacity cDNA Reverse Transcription Kit (Life Technologies). Expression of glyceraldehyde-3-phosphate dehydrogenase (GAPDH), beta actin (ACTB), and hypoxanthine phosphoribosyltransferase 1 (HPRT1) was analyzed by Quantitative Real-Time PCR using primers listed in S2 Table. Quantitative Real-Time PCR was performed using StepOne Real-Time PCR System and Power SYBR^®^ Green PCR Master Mix (Life Technologies). The cDNA equivalent to 5 ng of the large RNA fraction was used as template in each PCR reaction. As an additional precaution, all of the RNA samples were DNase-treated and the resulting RNA was tested with PCR to confirm the absence of DNA contamination. The thermal profile consisted of 1 holding stage of 95°C for 10 minutes and 40 cycling stages of 95°C for 15 seconds followed by 60°C for 1 minute. Melt curves were generated after 40 cycles of amplification. Expression of GAPDH, ACTB, and HPRT1 genes were assessed relative to the standard curves. Standard curves were generated using ACTB primers and control cDNA template ranging from 10 ng– 0.64 pg equivalents of the large RNA fraction. The use of DNA template as a universal external standard in qPCR allows for the assessment of differences in the transcript levels in RNA derived from different samples [12].

### Statistical analysis

Measured quantities for each sample were subjected to standard statistical analysis and the results are presented in the form of mean ± 1 standard deviation. To evaluate correlations among variables of interest, software packages R and Matlab [13] were used to perform robust linear regression analysis using Tukey’s bisquare M-estimator [14]. The software system Matlab was used as the primary tool, but the numerical accuracy of a subset of regression lines was validated by performing the identical test in the software package R. An identical process for measuring correlations between the variable of interest (total RNA), and other selected variables was also performed. In addition, the sums, products and ratios of various sets of variables were calculated and the new aggregate variables were used for subsequent analysis. Regression analysis between the aggregate variable and the variable of interest (e.g. total RNA) was performed and adjusted R-squared values were recorded. When appropriate, physical constraints were enforced—for example, to require that the intercept of the correlation line to pass through the origin (0,0) (e.g. zero amounts of the independent variable should result in zero quantities of total RNA).

For small and selected subsets of data, for which the standard parametric approach may result in statistical bias, we performed Monte Carlo sampling (resampling) to estimate the variability of means [15, 16]. This approach is non-parametric [17] and it does not use the structure of the model in resampling—the data are directly resampled as depicted in S2, S3 and S4 Figs.

## Results

### Blood RNA level and its inter-individual differences in healthy humans

In order to evaluate inter-individual differences in peripheral blood RNA level, blood samples were collected from 35 healthy donors, (25 females and 10 males). The collected blood samples were used for CBC analysis and for RNA and DNA isolation. Table 1 summarizes data on total RNA and DNA levels, and WBC counts in human peripheral blood. The complete data set is presented in the S1 Table. The mean total RNA level was 14.58 ± 4.47 μg/ml of blood, with a range of 6.69 to 22.72 μg/ml of blood. The CV of total RNA level for the 35 samples was 30.7. There was no significant difference (P>0.05) between the average total RNA level in blood from females (13.91 ± 4.41 μg/ml) and males (16.24 ± 4.41 μg/ml) in this sample group. The results showed a maximum of 3.4-fold inter-individual difference in the RNA level in peripheral blood of healthy individuals. The average amount of DNA was 52.77 μg/ml of blood, and the average WBC count was 6.96 x 10^6^ cells/ml of blood. In the studied group, no correlation between total RNA level and age was observed (Adjusted R-Square = 0.015, S1 Fig).

**Table 1:** Total RNA, DNA and White Cell Content in Human Blood.

	Age (years)	Total RNA (μg/ml blood)	DNA (μg/ml blood)	White Cells (μg/ml bood)
**Mean**	**62.63 ± 9.15**	**14.58 ± 4.47**	**52.77 ± 20.75**	**6.96 ± 2.54**
**Range**	**50–89**	**6.70–22.72**	**26.4–123.4**	**3.4–15.6**

Two vials of venous blood were collected from each of 35 individuals (25 females and 10 males). One vial of blood was used for the CBC analysis and the other was mixed with RNAzol^®^ BD for the isolation of RNA and DNA (Methods). The data are summarized as mean ± 1SD for each parameter.

### Distribution of large and small RNA fractions in human blood

**Fig 2:**
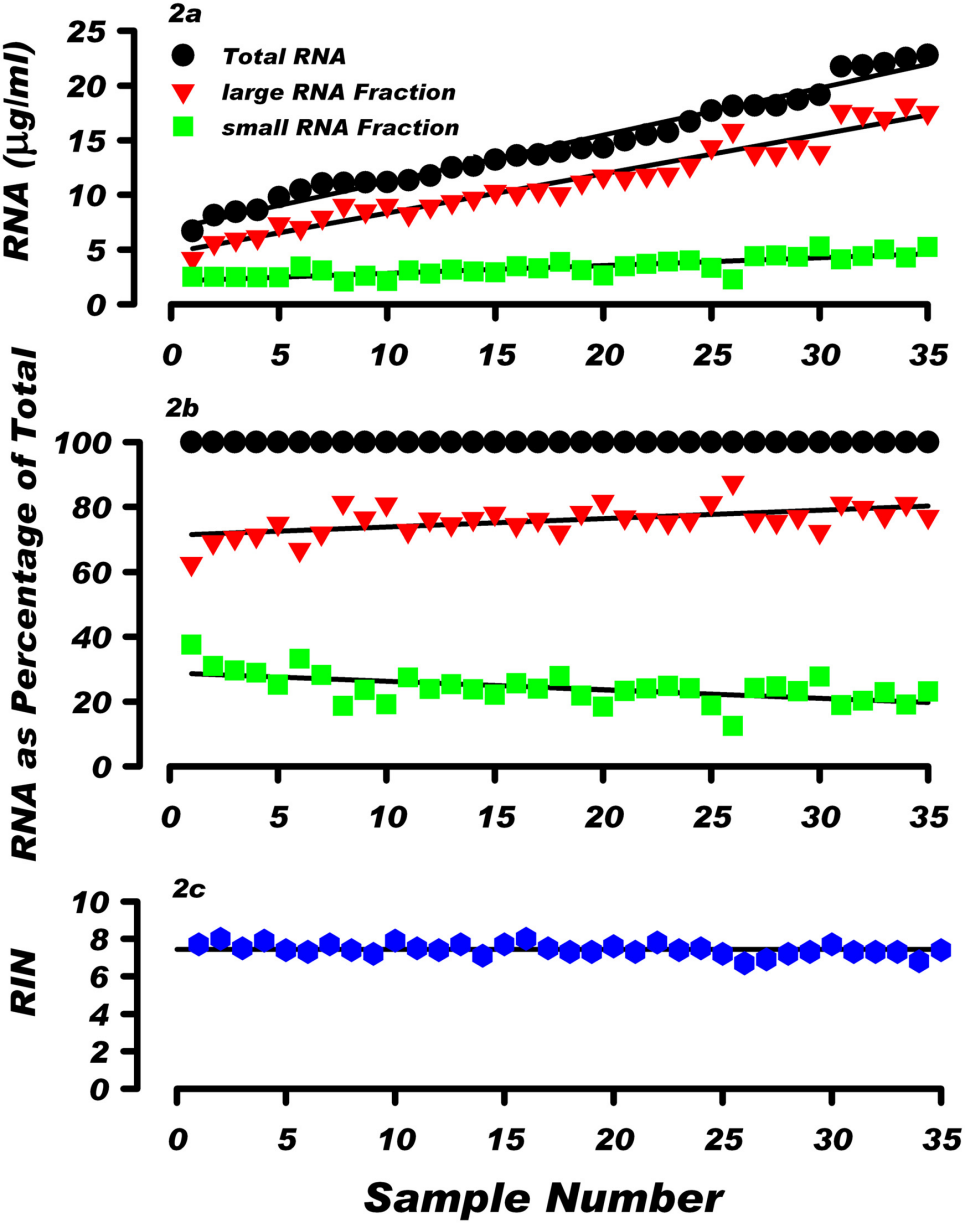
Characteristics of RNA isolated from healthy donors. a) Proportion of the large and small RNA fractions in relation to increasing quantities of total RNA in human peripheral blood. Samples of RNA from the 35 individuals are sequentially ranked in accord with the increasing amount of total RNA in the sample. For each sample, the amounts of the large RNA fraction (inverted triangle) and small RNA fraction (square) are depicted. b) The large RNA and small RNA fractions are expressed as a percentage of the total RNA in the sample. c) Bioanalyzer RIN values of the large RNA fractions from 35 samples.

The RNAzol^®^ BD protocol enables blood RNA to be fractionated into large RNA (>200 nucleotides) and small RNA (<200 nucleotides) fractions. Over 90% of the large RNA fraction is rRNA and the rest is mRNA and lncRNA [18]. The small RNA fraction contains tRNA, 5S rRNA, and small regulatory RNA including micro RNA. Characteristics of the isolated RNA and the distribution of large and small RNA fractions are illustrated in Fig 2. Fig 2a shows the amounts of the large RNA and small RNA fractions in relation to total RNA. The blood samples from 35 donors are rank-ordered according to increasing concentrations of total RNA. The amount of the large RNA and small RNA in human blood varies from 4.18 to 18.18 μg and 1.91 to 5.29 μg RNA/ml, respectively. When the quantities of large RNA and small RNA are expressed as a percentage of the total RNA (Fig 2b), the proportions of large and small RNA fractions are relatively constant in all 35 samples and amount to 75.84% ± 4.69% and 24.16% ± 4.69% of total RNA, respectively. Fig 2c shows that there was no significant difference in the integrity of the isolated RNA, with an average RIN of 7.43 ± 0.31.

### Electrophoretic profiles of blood RNA

**Fig 3:**
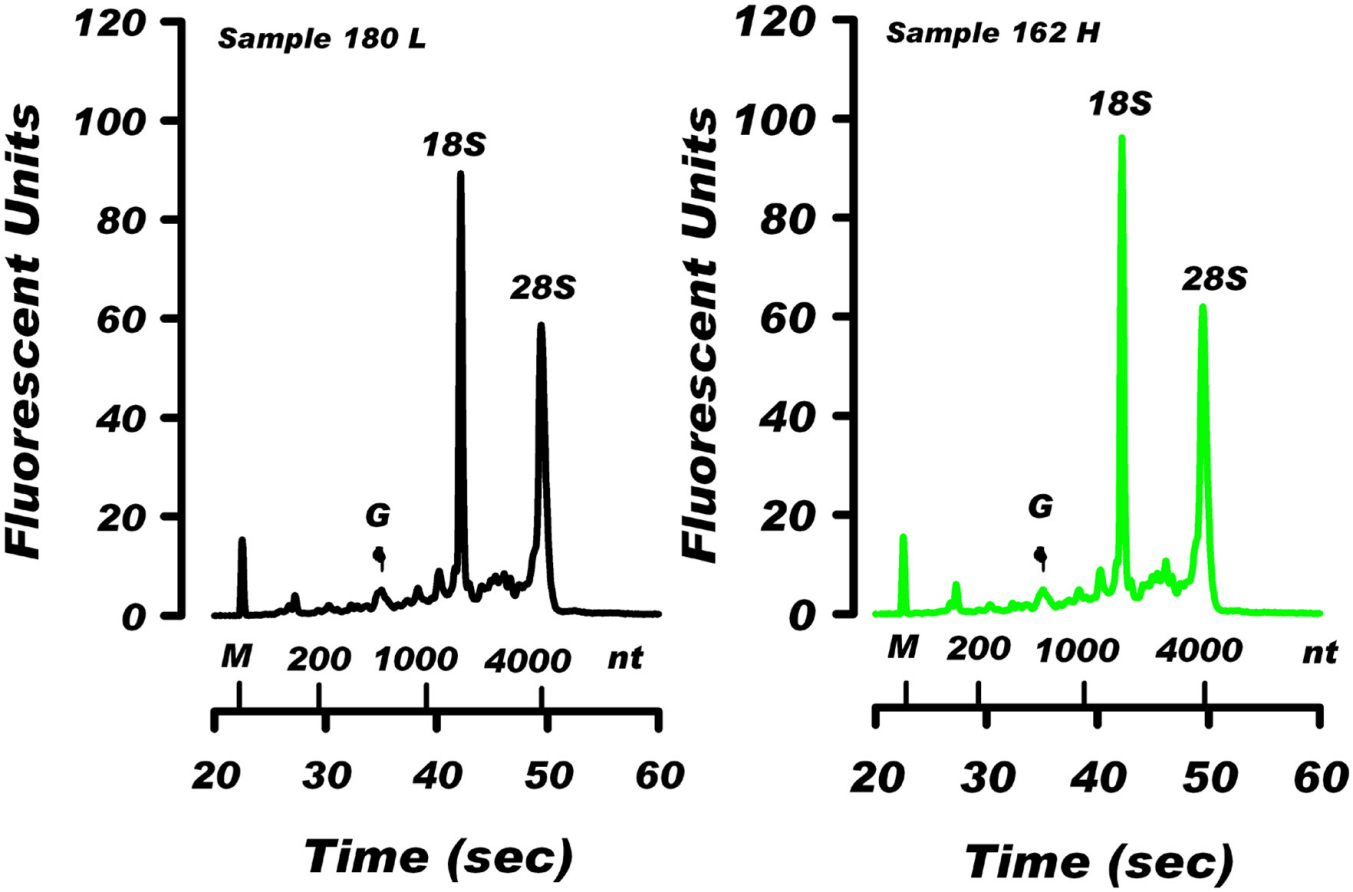
Bioanalyzer profiles of the large RNA fractions isolated from donors with the lowest and highest blood RNA level. The large RNA fractions (200 ng) from the low RNA level sample 180 (6.7 μg RNA/ml blood) and the high RNA level sample 162 (22.7 μg RNA/ml blood) were separated using the Bioanalyzer RNA Nano 6000 Kit. Shown are positions of: the (18S) and (28S) ribosomal RNA; the 600 nt globin region (G); and Bioanalyzer marker (M).

The Bioanalyzer electrophoretic profiles of RNA from all 35 samples showed a similar distribution of rRNA in samples with high and low RNA levels. This is illustrated in Fig 3 showing the profiles of the large RNA fractions derived from the low RNA level sample 180 (6.7 μg RNA/ml blood) and the high RNA level sample 162 (22.7 μg RNA/ml blood). The Bioanalyzer profiles and the ratio of rRNAs from both samples are similar. In addition, the RNA resolution profiles depicted in Fig 3 show a distinct peak at the 600 nucleotide (nt) region. This peak is seen in all 35 blood samples in this study, as well as in the data presented in our previous report [10] and in RNA derived from erythrocytes [19]. The position of the 600 nt peak corresponds to mRNAs for globin transcripts HBA (576 nt) and HBB (626 nt). This peak is eliminated after mRNA globin depletion (unpublished observation).

Electrophoretic patterns of blood RNA samples shown in Fig 3 indicate that the ratio of large (28S) rRNA to small (18S) rRNA is 1.02 and 1.06 for sample 180 and 162, respectively. For the group of 35 samples the average 28S/18S rRNA ratio is 0.96 ± 0.02 (not shown). This 28S/18S rRNA ratio is smaller than the ratio of 1.7–2.0 normally seen in most human tissues. This lower 28S/18S rRNA ratio represents an electrophoretic profile that may be characteristic of RNA isolated from human whole blood. Similar electrophoretic profiles were reported in the recent review of methods for RNA isolation from human whole blood [9]. By comparison, in our experiments (not shown) with RNA isolated from peripheral blood mononuclear cells the 28S/18S rRNA ratio was consistently > 1.9.

### Factors contributing to the inter-individual differences in blood RNA level

**Fig 4:**
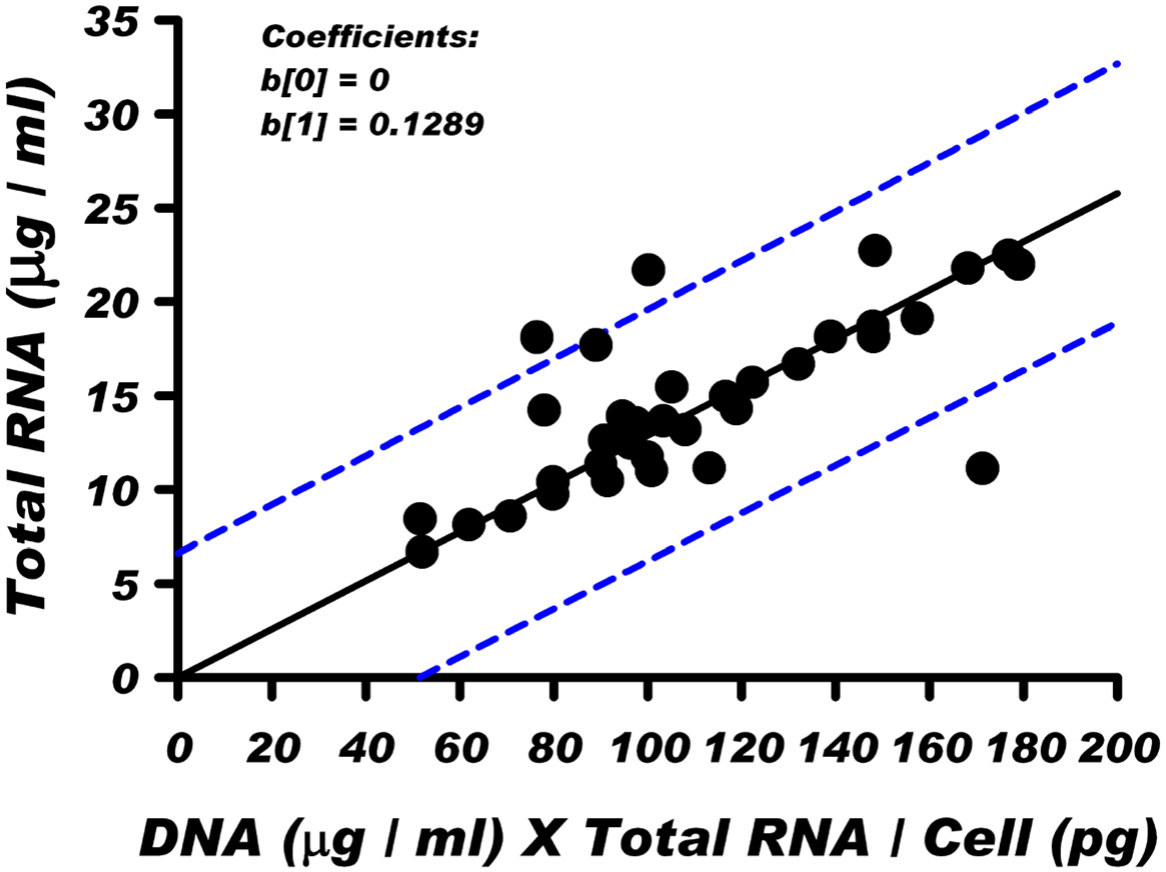
Robust regression analysis of total RNA vs the product of blood DNA level (μg DNA / ml blood) and cellular RNA content (pg RNA/ cell). The blue dashed line represents the 95% prediction interval for the group of 35 samples. An adjusted R-square value for this regression analysis is 0.947.

To identify factors in human blood that contribute to total RNA level, we performed statistical correlation analysis on the data obtained from the full set of 35 samples (S1 Table). In each case the statistical protocol tested for outliers (robust regression) and performed appropriate corrections to obtain adjusted R-squared values as noted in the methods description. As expected, both large RNA and small RNA fractions were strongly correlated to total RNA (adjusted R-squared = .974 and .712 respectively) but since total RNA levels are dependent on these two parameters, the goodness of fit as determined by R-squared, serves as a positive control for subsequent comparisons. First order correlations of total RNA with a single factor variable such as WBC number, monocytes, neutrophils, lymphocytes, μg DNA/ml blood and pg RNA/cell all resulted in moderate to weak adjusted R-square values: 0.622, 0.559, 0.254, 0.217, 0.049 and 0.004 respectively. Further evaluation using second order additive interactions did not identify sufficient improvement of adjusted R-squared values. However, a highly significant correlation was identified in a subsequent analysis when the total RNA level was correlated against the multiplicative interaction of two factors, the blood DNA level and the RNA content per WBC. As presented in Fig 4, this statistical analysis yielded a correlation with (adjusted) R-squared value of 0.947.

In order to further examine factors that may contribute to the 3.4-fold inter-individual differences in the blood RNA level, we selected for analysis two subpopulations (n = 5) at the extremes of the blood RNA level. We analyzed these samples with respect to: RNA and DNA levels, and WBC counts, neutrophil, lymphocyte, monocyte, RBC and platelet cell numbers (Table 2). Eosinophils and basophils as minor contributors to the WBC count are not shown here (the complete set of data is in S1 Table). The average RNA levels for the low and high RNA sample groups were 8.46 μg and 22.13 μg/ml of blood, respectively. This amounts to a 2.6 fold difference in the average RNA level in blood from these two sample groups. The high RNA level group had a 1.9 fold-higher WBC count and 2.1 fold-higher neutrophil count compared to the group with a low RNA blood level. The number of lymphocytes and monocytes in the high RNA group was about 1.6 fold higher than in the low RNA samples. The RBC and platelet counts were similar in the two groups.

**Table 2:** Total RNA Levels and Cell Counts in Individuals with Low and High Blood RNA Concentrations.

	Sample	RNA	RNA	DNA	WBC	Neutrophils		Lymphocytes		Monocytes		RBC	Platelet
	Number	μg/ml	pg/cell	μg/ml	10^3^/μl	10^3^/μl	%	10^3^/μl	%	10^3^/μl	%	10^6^/μl	10^3^/μl
**LOW RNA**	**180**	**6.69**	**1.97**	**26.44**	**3.4**	**1.9**	**55.9**	**1.1**	**32.4**	**0.3**	**8.8**	**4.12**	**268**
	**154**	**8.12**	**1.89**	**32.81**	**4.3**	**2.3**	**53.5**	**1.5**	**34.9**	**0.3**	**7.0**	**4.53**	**224**
	**168**	**8.43**	**1.76**	**29.36**	**4.8**	**2.9**	**60.4**	**1.4**	**29.2**	**0.3**	**6.3**	**3.99**	**226**
	**173**	**8.58**	**1.56**	**45.34**	**5.5**	**3.7**	**67.3**	**2.4**	**43.6**	**0.4**	**7.3**	**4.16**	**243**
	**163**	**10.47**	**1.90**	**48.30**	**5.5**	**2.8**	**50.9**	**2.0**	**36.4**	**0.5**	**9.1**	**4.47**	**222**
**Mean**		**8.46**	**1.82**	**36.45**	**4.7**	**2.7**	**57.6**	**1.7**	**35.3**	**0.4**	**7.7**	**4.25**	**237**
**SD**		**1.35**	**0.16**	**9.79**	**0.9**	**0.7**	**6.4**	**0.5**	**5.4**	**0.1**	**1.2**	**0.23**	**19**
**HIGH RNA**	**162M**	**22.72**	**2.77**	**53.56**	**8.2**	**5.5**	**67.1**	**2.0**	**24.4**	**0.4**	**4.9**	**4.42**	**250**
	**152M**	**22.46**	**2.64**	**66.93**	**8.5**	**5.1**	**60.0**	**2.4**	**28.2**	**0.7**	**8.2**	**4.63**	**265**
	**150**	**21.99**	**1.90**	**94.44**	**11.6**	**7.7**	**66.4**	**2.4**	**20.7**	**0.8**	**6.9**	**3.85**	**342**
	**153**	**21.78**	**3.07**	**54.80**	**7.1**	**4.6**	**64.8**	**1.9**	**26.8**	**0.4**	**5.6**	**4.48**	**257**
	**151**	**21.69**	**2.24**	**44.80**	**9.7**	**5.8**	**59.8**	**2.6**	**26.8**	**0.8**	**8.2**	**4.85**	**317**
**Mean**		**22.13**	**2.52**	**62.91**	**9.0**	**5.7**	**63.6**	**2.3**	**25.4**	**0.6**	**6.8**	**4.45**	**286**
**SD**		**0.45**	**0.46**	**19.31**	**1.7**	**1.2**	**3.5**	**0.3**	**3.0**	**0.2**	**1.5**	**0.37**	**40.85**
**Fold increase**		**2.62**	**1.39**	**1.73**	**1.9**	**2.1**	**1.1**	**1.3**	**0.7**	**1.7**	**0.9**	**1.0**	**1.2**

Data from five individuals with the lowest and highest levels of RNA are depicted along with their respective DNA level and CBC analysis as described in Methods. Shown are means, SD, and fold increase for each parameter. M, male donors.

As expected, blood DNA levels shown in Table 2 parallel changes in the white cell number. The average cellular RNA content was 1.82 and 2.52 pg per cell for the low- and high-RNA groups, respectively. This amounts to 1.4-fold increase in the cellular RNA content in the group with the higher RNA blood level. Thus, similar to the robust statistical correlation analysis of all 35 samples, comparison of the low- and high-RNA level samples indicate that the elevated RNA level in human peripheral blood may result from an interplay of two factors, an increase in white cell number and an increase of the RNA content in the white blood cells.

### Comparison of gene expression in blood samples with low and high RNA level

In the evaluation of gene expression by qPCR it is a common approach to use samples containing a fixed amount of RNA. This approach, however, does not take into account variable levels of RNA in blood derived from various donors. To determine how low- and high-blood RNA levels affect quantitation of gene expression, we quantitated housekeeping gene mRNAs in blood samples previously described in Table 2. The quantitation comprised three housekeeping genes: glyceraldehyde 3-phosphate dehydrogenase (GAPDH), beta actin (ACTB) and hypoxanthine phosphoribosyltransferase 1 (HPRT1). The data are summarized in Table 3. When the average mRNA level was calculated per ng of RNA used for reverse transcription, both low and high RNA level samples had similar amounts of mRNA for GAPDH, ACTB and HPRT1. The data analysis employing the resampling approach for equality of means demonstrated that the means in either the high or low RNA groups were not different at a statistically significant level (Table 3, and S2–S4 Figs). However when the average mRNA level was expressed in units per ml of blood, the high-RNA level samples had 2.44, 2.34 and 1.85 fold higher amounts of mRNA for GAPDH, ACTB and HPRT, respectively, than the low RNA level samples. Furthermore, with the gene activity expressed as units per ml of blood, differences in expression levels reached the significance level (P<0.05) (Table 3 and S2–S4 Figs). These calculations indicate that variability is a very common feature of RNA expression data, and normalization is fundamental to an effective understanding of the data. Moreover, the approach to normalization should account for the manner in which mRNA amounts are expressed, as the units of data have a substantial impact on the statistical outcome and the physiological conclusion that is reached.

**Table 3:** Normalized Gene Expression in Individuals with Low and High Blood RNA Levels.

RNA	Sample	Total RNA	GAPDH		ACTB		HPRT	
Sample Group	Number	(μg/ml blood)	(U/ng RNA)	(U/ml blood x 10^3^)	(U/ng RNA)	(U/ml blood x 10^3^)	(U/ng RNA)	(U/ml blood x 10^3^)
**LOW**	**180**	**6.69**	**14.31**	**95.72**	**208.25**	**1393.19**	**0.94**	**6.31**
	**154**	**8.12**	**12.90**	**104.72**	**154.67**	**1255.91**	**0.59**	**4.82**
	**168**	**8.43**	**12.70**	**107.10**	**288.19**	**2429.40**	**0.80**	**6.76**
	**173**	**8.58**	**8.91**	**76.44**	**288.08**	**2471.75**	**0.97**	**8.32**
	**163**	**10.47**	**12.05**	**126.12**	**143.92**	**1506.83**	**0.68**	**7.08**
	**Mean**	**8.46**	**12.17**	**102.02**	**216.62**	**1811.42**	**0.80**	**6.66**
	**SD**	**1.35**	**2.00**	**18.08**	**69.68**	**590.38**	**0.16**	**1.27**
**HIGH**	**162M**	**22.72**	**12.98**	**294.89**	**182.21**	**4139.79**	**0.55**	**12.59**
	**152M**	**22.46**	**11.80**	**265.06**	**165.87**	**3725.45**	**0.54**	**12.18**
	**150**	**21.99**	**5.83**	**128.12**	**140.09**	**3080.56**	**0.36**	**7.98**
	**153**	**21.78**	**6.40**	**139.31**	**141.38**	**3079.18**	**0.37**	**7.99**
	**151**	**21.69**	**19.25**	**417.47**	**330.33**	**7164.78**	**0.96**	**20.77**
	**Mean**	**22.13**	**11.25**	**248.97**	**191.97**	**4237.95**	**0.56**	**12.30**
	**SD**	**0.45**	**5.48**	**149.55**	**79.33**	**1697.13**	**0.24**	**5.22**
**Probability Test**			**NS**	**P< 0.05**	**NS**	**P< 0.05**	**NS**	**P< 0.05**
**Fold Increase**		**2.62**	**0.92**	**2.44**	**0.89**	**2.34**	**0.70**	**1.85**

RNA from subpopulations of individuals with the lowest and highest levels of RNA was used to characterize gene expression by RT-qPCR as described in Methods. The amounts of GAPDH, ACTB and HPRT1 transcripts were quantitated in units per ng of RNA used for RT-qPCR and in units per ml of blood. Shown are means, ± 1 SD, and a summary of the resulting probability tests based on non-parametric Monte Carlo sampling [17]. M, male donors.

In addition, it is important to note that the results presented in Table 3 also show substantial inter-individual differences in expression of the selected housekeeping genes. The mRNA level expressed per ng of total RNA ranges from 5.8 to 19.3 units for GAPDH, 140 to 330 units for ACTB and 0.36 to 0.97 units for HPRT1. Similarly, the mRNA level expressed per ml of blood ranges from: 76 to 417, 1256 to 7165 and 4.8 to 20.8 units for GAPDH, ACTB and HPRT1, respectively. Thus, levels of the three transcripts showed 2.4- to 3.3- fold inter-individual differences when expressed per ng of total RNA, and 4.3- to 5.7-fold inter-individual differences when expressed per ml of blood.

## Discussion

The analysis of gene expression in human peripheral blood is considered to be an attractive resource for assessing the metabolic and pathological status of a blood donor. To monitor gene expression in peripheral blood, it is important to consider ex vivo RNA degradation and the effectiveness of RNA recovery from the blood. It was shown that some RNA transcripts are relatively stable but hundreds of RNA transcripts are sensitive to ex vivo room temperature storage before RNA extraction [6, 20, 21]. In order to preserve the in vivo gene expression patterns in our study, blood was mixed with RNAzol^®^ BD immediately after the blood draw. Processing of blood in RNAzol^®^ BD allows for quantitative recovery of RNA [10]. Quantitative recovery of blood RNA has not been sufficiently addressed in previous studies. Many publications do not provide data regarding the recovery of RNA from blood while others report a recovery of only 1 to 6 μg of total RNA per ml of human blood [5–9]. In the current study, we determined that the average total RNA level in peripheral blood of 35 females and 10 males, age 50 to 89 years, was 14.58 μg per ml of blood (Table 1 and S1 Table). There was no statistical difference in the amount of RNA recovered from blood of female and male subjects.

The total RNA level in blood from various individuals ranged from 6.7 to 22.7 μg per ml of blood. The 3.4-fold inter-individual differences in total RNA level in blood from healthy donors are documented in Figs 1, 2a and Table 1. The CV for total RNA for the 35 samples was 30.7. For comparison, the results in Fig 1 show that in healthy donors the blood RNA level, whether low or high was stable, with a mean CV for intra-individual variation of 5.87%. Thus, CV for inter-individual variation is 5.2 fold higher than CV for intra-individual variation. In addition, the CV associated with repeated RNA extraction from the same sample was only 2.74 (Methods). These data demonstrate that intra-individual fluctuations and methodological variability cannot explain the 3.4 fold range of inter-individual blood RNA levels observed among the 35 donors in this study.

The analysis of all 35 samples (S1 Table) and the data presented in Figs 2 and 3 indicate that the large RNA fraction comprises about 75% of total RNA in both, the low- and high RNA level samples. Since this fraction contains predominantly rRNA (Fig 3) it suggests that rRNA is the most significant factor contributing to the inter-individual differences in the total RNA level in human blood. The ribosomal 18S RNA and 28S RNA constitute over 75% of the cellular RNA (Fig 2b) [10, 18]. The inter-individual variability of rRNA content in blood may reflect variability in the transcription of clusters of ribosomal genes (rDNA). The cluster lengths of rDNA in the human peripheral WBC genome is known to range from 50 kb to >6 Mb [22]. Variability in rRNA accumulation has also been linked to pathological changes in human cells. Human rDNA clusters are known to be recombinational hotspots in cancer [23] and overexpression of rRNA is common in prostate cancer [24]. Impaired ribosome biogenesis and function have been implicated in several human diseases [25]. Reduced expression of rRNA may contribute to defective hematopoesis in patients with myelodysplastic syndrome [26]. Since our study involved healthy subjects, the pathological and physiological consequences of the observed variations in blood RNA level, and the contribution of the various blood cell types to these variations remain to be investigated.

The average RNA level of 14.58 μg per ml of human blood documented in our study indicate that in previous studies only a small portion (15–26%) of blood RNA was extracted and analyzed for gene expression. It is unknown whether lower-yield extractions proportionally recover all lncRNA and mRNA species that are present in whole blood. It is of interest that electrophoretic RNA profiles presented in this report (Fig 3) and in previous studies [10, 19] show the presence of a 600 nucleotide peak, corresponding in size, to the globin mRNAs. This peak is missing in the electrophoretic profiles of RNA extracted from human blood with low yields [7, 9]. This may indicate that low-yield extractions result in RNA preparations with a significantly reduced proportion of shorter mRNA and lncRNA transcripts [27].

The extent to which RNA levels in other human tissues parallel inter-individual variations of the RNA level in blood is not known. Our observations with animal tissues indicate that the 3–4 fold differences in the blood RNA level is likely limited to blood. Over many years, we have performed numerous RNA extractions of rat and mouse tissues including: liver, muscles, kidney and brain derived from dozens of animals. The amount of RNA isolated from tissues of healthy animals was relatively unchanged and variations in the RNA level per mg of tissue were within the limit of ± 15% (unpublished observation). It is of interest to note that a recent study using rat whole blood reported a large inter-animal variation (5-fold) in the amount of total RNA extracted from blood (12.5–62.5 μg RNA /ml, [28]).

The diversity of factors that may contribute to the inter-individual differences of total RNA level in blood is reflected by the fact that, among the measured variables in this study (S1 Table), there was no strong correlation between total RNA level and a single blood component. The strongest correlation (adjusted r-square 0.947) was achieved when total RNA level was plotted (Fig 4) against the product of the blood DNA level (μg DNA / ml) and cellular RNA content (pg / cell). It is reasonable to expect that changes in total RNA level would correlate with fluctuations in cell number and changes in the RNA content of cells.

The complex relationship between inter-individual differences in the blood RNA level and blood components is also illustrated in Table 2 showing comparisons of blood samples with low and high RNA levels. Data in Table 2 show that, similar to the robust statistical correlations of the 35 samples (Fig 4), the blood RNA level is influenced by the number of WBC’s and the amount of RNA per WBC. The interplay of these two factors contributes to the differences in RNA levels observed in blood derived from different subjects. Determination of the cell type(s) that may contribute to the observed differences in the blood RNA levels will require quantitation of RNA levels within subpopulations of blood cells. Since neutrophils constitute about 60% of WBCs in human blood, the inter-individual differences in the RNA blood levels observed in our study may reflect, in part, the contribution of this group of blood cells.

It remains to be investigated if there are physiological consequences of the low or high levels of RNA in the circulation. However, our results indicate that the level of RNA in blood is an important parameter for quantitation of gene expression. Currently, the most commonly used denominator for calculating gene expression in blood is the quantity of total RNA or large RNA fraction used for analysis. The data presented in Figs 1 and 2; Tables 2 and 3 show that due to the inter-individual differences in RNA level, a fixed amount of RNA, used in gene expression studies as a denominator, does not account for the variable quantity of total RNA in blood derived from different individuals. To account for these inter-individual differences in the blood RNA level, the quantitation of transcripts in blood should include quantitating the amount of transcript per fixed amount of RNA as well as quantitating the amount of transcript per ml of blood. Previously, transcripts for housekeeping genes such as: GAPDH, beta-actin and beta 2-microglobulin (B2M) were used as internal standards to normalize the results of gene expression in blood. However, the usefulness of this approach was challenged due to significant inter-individual variations in the levels of housekeeping gene transcripts. This was observed in our studies (Table 3) and was reported previously [29–32]. Thus, due to the variable amount of RNA in blood, a comprehensive interpretation of gene expression results requires quantitation of gene activities per unit of RNA and per ml of blood.

Similar to RT-qPCR, the inter-individual differences in blood RNA level may affect quantitation of mRNA in RNA sequencing. In RNA sequencing, a fixed amount of total RNA or the large RNA fraction containing rRNA and mRNA is used for analysis. The rRNA- and globin-depleted RNA is converted to cDNA and amplified, and an equal amount of amplified cDNA is used for sequencing [5, 18, 33]. The sequencing results are presented as mapped reads per length of transcript in kilobase per million mapped reads (RPKM) or per number of fragments per length of transcript in kilobase per million mapped fragments (FPKM). A normalization approach relying only on RPKM or FPKM allocated to a fix amount of cDNA does not account the 3.4 fold inter-individual differences in the blood RNA level reported in our study.

The significance of taking into account the blood RNA level in the interpretation of gene expression data is illustrated in Table 3 with the analysis of blood samples with low and high RNA levels. Both groups of samples have similar GAPDH, ACTB and HPRT1 transcript levels when the data are expressed per ng of RNA. However, when gene transcript levels are computed per ml of blood, statistically significant differences are observed between these two groups. Therefore, the circulating level of gene transcripts in the blood may be an important factor to consider for interpretation of the physiological significance of gene expression studies. Decades of physiological and pharmacological research have demonstrated that the blood level of a compound is a critically important factor for evaluation of its biological effects. The absence of data on the RNA level in blood precludes calculations of the blood level of a studied gene transcript(s). Consequently, this may lead to an incomplete interpretation of study results.

## Supporting Information

S1 Fig Total Blood RNA concentrations plotted in relation to the age of the donor. The red circles and blue inverted triangles represent the female and male donors in the sample group. The blue dashed line represents the 95% prediction interval for the sample. A cluster of 12 (34%) individuals ranging in age from 55–60 years of age display mean blood RNA concentrations that span the entire range of values reported in this study.
(EPS)

S2 Fig Test for equality of GAPDH gene expression when the transcript is normalized as U / ng RNA or U / ml blood and the equality of means is tested using the bootstrap procedure. The Null-hypothesis is rejected if any of the means (red dot) lie outside the decision line (green).
(TIF)

S3 Fig Test for equality of ACTB gene expression when the transcript is normalized as U / ng RNA or U / ml blood and the equality of means is tested using the bootstrap procedure. The Null-hypothesis is rejected if any of the means (red dot) lie outside the decision line (green).
(TIF)

S4 Fig Test for equality of HPRT1 gene expression when the transcript is normalized as U / ng RNA or U / ml blood and the equality of means is tested using the bootstrap procedure. The Null-hypothesis is rejected if any of the means (red dot) lie outside the decision line (green).
(TIF)

S1 Table Summary of the RNA, DNA and complete blood cell analysis for 35 healthy male and female donors. M, male donors.
(DOCX)

S2 Table The forward and reverse primer sequences utilized for gene expression analysis. (XLSX)
