# Supplementary figures and images for: Inter-Individual Differences in RNA Levels in Human Peripheral Blood
Source: PLoS One. 2016 Feb 10;11(2):e0148260. doi: 10.1371/journal.pone.0148260 (PMC4749217; doi:10.1371/journal.pone.0148260)

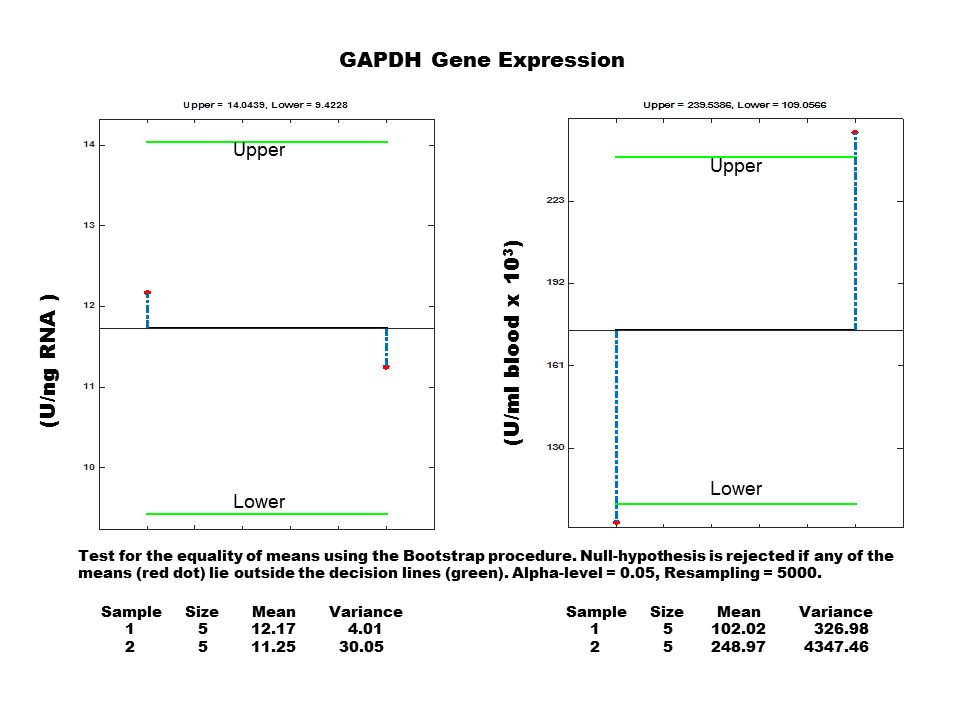

Supplement: S2 Fig — The Null-hypothesis is rejected if any of the means (red dot) lie outside the decision line (green). (TIF) [file pone.0148260.s002.tif]

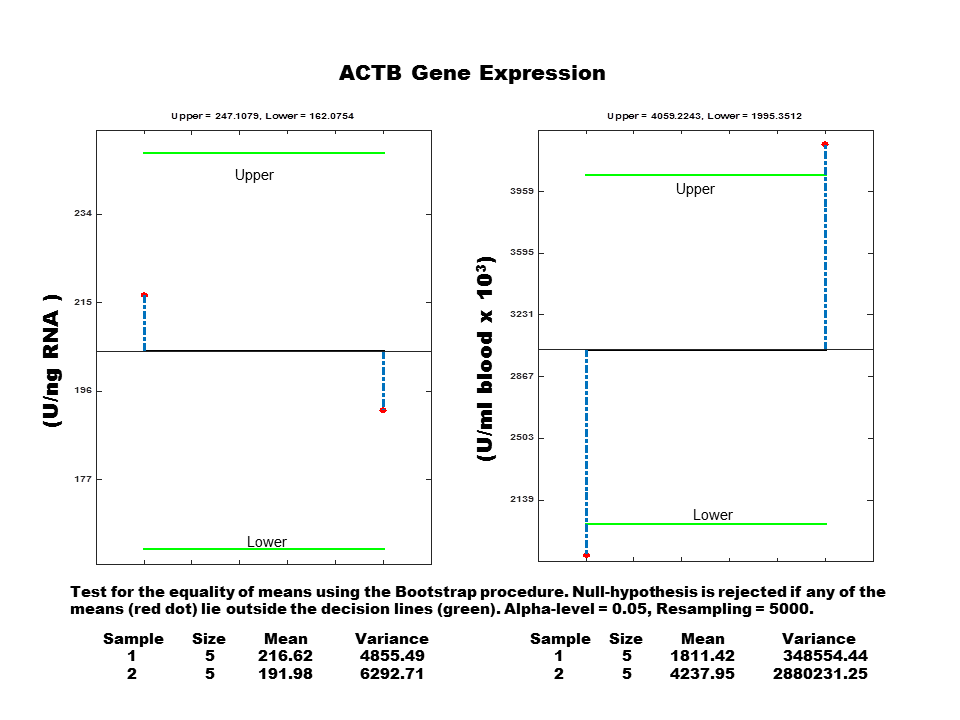

Supplement: S3 Fig — The Null-hypothesis is rejected if any of the means (red dot) lie outside the decision line (green). (TIF) [file pone.0148260.s003.tif]

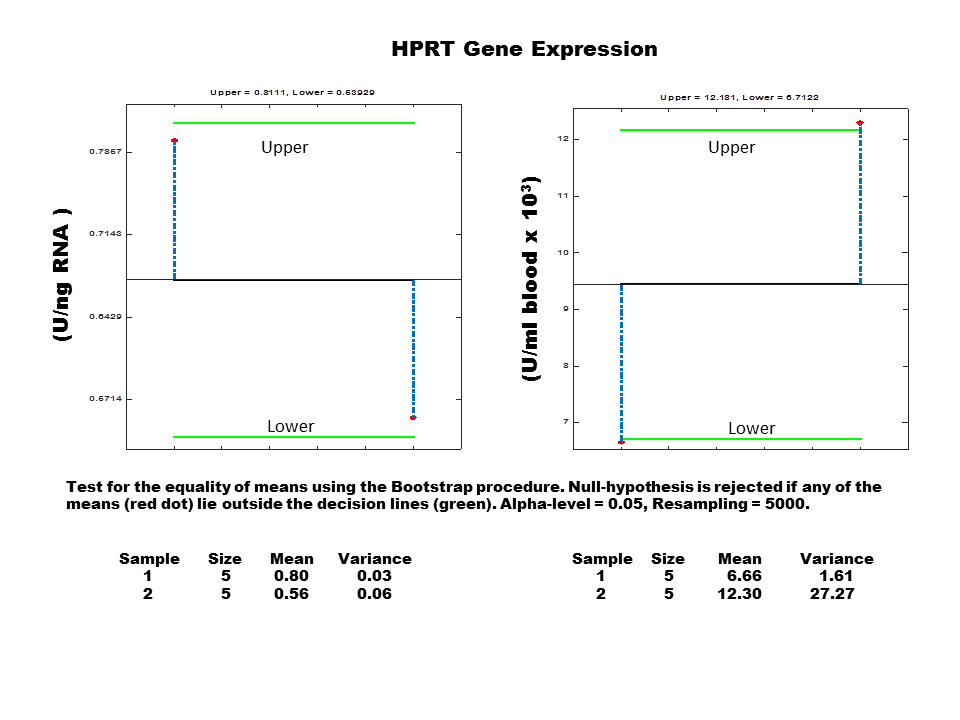

Supplement: S4 Fig — The Null-hypothesis is rejected if any of the means (red dot) lie outside the decision line (green). (TIF) [file pone.0148260.s004.tif]
