# Supplementary material for: Inter-Individual Differences in RNA Levels in Human Peripheral Blood
Source: PLoS One. 2016 Feb 10;11(2):e0148260. doi: 10.1371/journal.pone.0148260 (PMC4749217; doi:10.1371/journal.pone.0148260)
Supplement: Supplementary file 2 [file pone.0148260.s005.docx]

## S1 Table. RNA, DNA and Complete Blood Cell Analysis of 35 Donors age 50-89 years

| Patient ID | Age | Ethnicity | Total RNA (µg/ml blood) | large RNA (µg/ml blood) | small RNA (µg/ml blood) | DNA (ug/ml blood) | WBC counts ($10^3$ µl blood) | pg RNA / cell | Neutrophils (x $10^3$ / µl blood) | Lymphocytes (x $10^3$ /µl blood) | Monocytes (x $10^3$ /µl blood) | Eosinophils (x $10^3$ /µl blood) | Basophils (x $10^3$ /µl blood) | Immature Granulocytes (x $10^3$ /µl blood) | RBC's ( x $10^6$ / µl blood) | Platelets ( x $10^3$ /µl blood) | Hematocrit (%) | RIN |
|---|---|---|---|---|---|---|---|---|---|---|---|---|---|---|---|---|---|---|
| 172 | 57 | Cau | 13.92 | 10.05 | 3.88 | 42.14 | 6.2 | 2.245 | 3.2 | 2.3 | 0.5 | 0.1 | 0 | 0 | 4.27 | 313 | 36.4 | 7.3 |
| 173 | 58 | Cau | 8.58 | 6.12 | 2.47 | 45.34 | 5.5 | 1.560 | 3.7 | 2.4 | 0.4 | 0.1 | 0.1 | 0 | 4.16 | 243 | 41.1 | 7.9 |
| 177M | 52 | Cau | 18.68 | 14.36 | 4.32 | 68.91 | 8.7 | 2.147 | 5 | 2.7 | 0.6 | 0.2 | 0 | 0 | 5.08 | 236 | 34.8 | 7.3 |
| 178 | 59 | Cau | 16.69 | 12.68 | 4.01 | 123.4 | 15.6 | 1.070 | 12.5 | 1.3 | 1.7 | 0 | 0 | 0 | 3.55 | 321 | 38.9 | 7.5 |
| 179 | 51 | Cau | 18.16 | 13.7 | 4.47 | 40.76 | 5 | 3.632 | 3.1 | 1.4 | 0.4 | 0.1 | 0 | 0 | 5.45 | 200 | 41.7 | 7.2 |
| 180 | 58 | Cau | 6.69 | 4.18 | 2.51 | 26.44 | 3.4 | 1.968 | 1.9 | 1.1 | 0.3 | 0.1 | 0 | 0 | 4.12 | 268 | 39.6 | 7.7 |
| 161 | 55 | Cau | 18.11 | 15.84 | 2.27 | 38.83 | 9.2 | 1.968 | 5 | 3.4 | 0.5 | 0.3 | 0.1 | 0 | 4.78 | 324 | 43.5 | 6.7 |
| 162M | 56 | Cau | 22.72 | 17.48 | 5.24 | 53.56 | 8.2 | 2.771 | 5.5 | 2 | 0.4 | 0.2 | 0.1 | 0.1 | 4.42 | 250 | 42.6 | 7.4 |
| 163 | 57 | Cau | 10.47 | 9.08 | 1.91 | 48.03 | 5.5 | 1.904 | 2.8 | 2 | 0.5 | 0.2 | 0 | 0 | 4.47 | 222 | 44 | 7.4 |
| 165 | 59 | Cau | 12.63 | 9.63 | 3 | 43.88 | 6.1 | 2.070 | 3.5 | 2 | 0.4 | 0.1 | 0 | 0 | 4.71 | 279 | 40 | 7.1 |
| 166 | 79 | Cau | 11.15 | 9 | 2.15 | 66.95 | 6.6 | 1.689 | 1.8 | 3.6 | 0.8 | 0.3 | 0.1 | 0 | 4.41 | 183 | 38.7 | 7.9 |
| 169 | 62 | Cau | 11.12 | 8.5 | 2.62 | 70.87 | 4.6 | 2.417 | 2.4 | 1.6 | 0.5 | 0.2 | 0 | 0 | 4.77 | 228 | 38.2 | 7.2 |
| 159 | 70 | IN/IT | 13.66 | 10.39 | 3.27 | 54.47 | 7.2 | 1.897 | 4.7 | 1.9 | 0.5 | 0.1 | 0 | 0 | 4.25 | 301 | 45.3 | 7.5 |
| 160M | 72 | Cau | 12.47 | 9.31 | 3.16 | 63.33 | 8.2 | 1.521 | 5 | 1.7 | 1.1 | 0.4 | 0 | 0 | 4.59 | 225 | 42.4 | 7.7 |
| 167M | 56 | Cau | 15.72 | 11.82 | 3.9 | 62.24 | 8 | 1.965 | 5 | 2.1 | 0.6 | 0.2 | 0.1 | 0 | 5.14 | 246 | 43.4 | 7.4 |
| 170 | 82 | AA | 11.72 | 8.92 | 2.8 | 42.63 | 5 | 2.344 | 2.3 | 2 | 0.3 | 0.4 | 0 | 0 | 4.16 | 185 | 41.6 | 7.4 |
| 171M | 67 | Cau | 11.01 | 7.92 | 3.1 | 50.36 | 5.5 | 2.002 | 2.8 | 1.6 | 0.7 | 0.3 | 0 | 0 | 4.67 | 202 | 42.3 | 7.7 |
| 101 | 71 | Cau | 17.69 | 14.36 | 3.32 | 39.74 | 7.9 | 2.239 | 5.3 | 1.5 | 0.6 | 0.4 | 0.1 | 0 | 4.42 | 294 | 40.9 | 7.2 |
| 151 | 63 | Cau | 21.69 | 17.6 | 4.1 | 44.8 | 9.7 | 2.236 | 5.8 | 2.6 | 0.8 | 0.5 | 0 | 0 | 4.85 | 317 | 40.6 | 7.3 |
| 157 | 64 | Cau | 14.23 | 11.13 | 3.11 | 34.52 | 6.3 | 2.259 | 2.9 | 2.8 | 0.4 | 0.2 | 0 | 0 | 4.25 | 233 | 39.7 | 7.3 |
| 158 | 68 | AA | 13.57 | 10.09 | 3.48 | 44.5 | 6.2 | 2.189 | 3.5 | 2.3 | 0.4 | 0 | 0 | 0 | 4.24 | 356 | 41.5 | 8 |
| 164M | 89 | His | 10.41 | 6.96 | 3.45 | 45.28 | 5.9 | 1.764 | 3.4 | 1.6 | 0.5 | 0.5 | 0 | 0 | 4.08 | 178 | 45.5 | 7.3 |
| 168 | 64 | AA | 8.43 | 5.93 | 2.49 | 29.36 | 4.8 | 1.756 | 2.9 | 1.4 | 0.3 | 0.1 | 0 | 0 | 3.99 | 226 | 47.2 | 7.5 |
| 93 | 65 | Cau | 13.19 | 10.28 | 2.91 | 42.56 | 5.2 | 2.537 | 3 | 1.8 | 0.4 | 0 | 0 | 0 | 4.65 | 253 | 33.6 | 7.7 |
| 94M | 65 | Cau | 15.47 | 11.76 | 3.71 | 48.93 | 7.2 | 2.149 | 3 | 3.2 | 0.7 | 0.3 | 0.1 | 0 | 5.1 | 255 | 41.8 | 7.8 |
| 96M | 74 | AA | 14.31 | 11.67 | 2.64 | 60.64 | 7.3 | 1.960 | 4.1 | 2 | 0.8 | 0.3 | 0 | 0 | 4.56 | 260 | 42.6 | 7.6 |
| 98 | 57 | Cau | 9.76 | 7.33 | 2.46 | 60.52 | 7.4 | 1.319 | 5.6 | 1.3 | 0.3 | 0.1 | 0 | 0 | 4.63 | 262 | 42 | 7.4 |
| 102M | 58 | Cau | 19.12 | 13.83 | 5.29 | 101.28 | 12.3 | 1.554 | 8.7 | 2.8 | 0.6 | 0.1 | 0 | 0 | 4.89 | 271 | 43.7 | 7.7 |
| 103 | 64 | Cau | 11.32 | 8.22 | 3.09 | 28.63 | 3.6 | 3.144 | 1.6 | 1.7 | 0.2 | 0.1 | 0 | 0 | 4.1 | 203 | 46.8 | 7.5 |
| 150 | 70 | Cau | 21.99 | 16.97 | 5.01 | 94.4 | 11.6 | 1.896 | 7.7 | 2.4 | 0.8 | 0.5 | 0.1 | 0.1 | 3.85 | 342 | 45.7 | 7.3 |
| 152M | 61 | Cau | 22.46 | 18.18 | 4.28 | 66.93 | 8.5 | 2.642 | 5.1 | 2.4 | 0.7 | 0.2 | 0 | 0 | 4.63 | 265 | 45 | 6.8 |
| 153 | 57 | Cau | 21.78 | 17.37 | 4.41 | 54.8 | 7.1 | 3.068 | 4.6 | 1.9 | 0.4 | 0.2 | 0 | 0 | 4.48 | 257 | 40.7 | 7.3 |
| 154 | 50 | Cau | 8.12 | 5.6 | 2.51 | 32.81 | 4.3 | 1.888 | 2.3 | 1.5 | 0.3 | 0.1 | 0 | 0 | 4.53 | 224 | 32.9 | 8 |
| 155 | 52 | Cau | 14.97 | 11.49 | 3.48 | 41.27 | 5.3 | 2.825 | 3 | 1.7 | 0.4 | 0.1 | 0 | 0 | 4.99 | 265 | 42.6 | 7.3 |
| 156 | 50 | AA | 18.15 | 13.76 | 4.39 | 33.68 | 4.4 | 4.125 | 2.2 | 1.8 | 0.3 | 0.1 | 0 | 0 | 4.62 | 287 | 39.4 | 6.9 |
| Mean | 62.63 | | 14.58 | 11.19 | 3.41 | 52.77 | 6.96 | 2.19 | 4.14 | 2.05 | 0.55 | 0.20 | 0.02 | 0.01 | 4.51 | 256.40 | 41.33 | 7.43 |
| ±1 SD | 9.15 | | 4.47 | 3.77 | 0.90 | 20.75 | 2.54 | 0.62 | 2.18 | 0.61 | 0.28 | 0.14 | 0.04 | 0.02 | 0.40 | 45.42 | 3.40 | 0.31 |
